# An adenylyl cyclase with a phosphodiesterase domain in basal plants with a motile sperm system

**DOI:** 10.1038/srep39232

**Published:** 2016-12-16

**Authors:** Masahiro Kasahara, Noriyuki Suetsugu, Yuki Urano, Chiaki Yamamoto, Mikiya Ohmori, Yuki Takada, Shujiro Okuda, Tomoaki Nishiyama, Hidetoshi Sakayama, Takayuki Kohchi, Fumio Takahashi

**Affiliations:** 1 Graduate School of Life Sciences, Ritsumeikan University, Shiga 525-8577, Japan; 2 College of Life Sciences, Ritsumeikan University, Shiga 525-8577, Japan; 3 Graduate School of Biostudies, Kyoto University, Kyoto 606-8502, Japan; 4 Graduate School of Medical and Dental Sciences, Niigata University, Niigata 951-8510, Japan; 5 Advanced Science Research Center, Kanazawa University, Ishikawa 920-0934, Japan; 6 Department of Biology, Graduate School of Science, Kobe University, Hyogo 657-8501, Japan

## Abstract

Adenylyl cyclase (AC), which produces the signalling molecule cAMP, has numerous important cellular functions in diverse organisms from prokaryotes to eukaryotes. Here we report the identification and characterization of an *AC* gene from the liverwort *Marchantia polymorpha*. The encoded protein has both a C-terminal AC catalytic domain similar to those of class III ACs and an N-terminal cyclic nucleotide phosphodiesterase (PDE) domain that degrades cyclic nucleotides, thus we designated the gene Mp*CAPE (*C*OMBINED *A*C with *P*D*E**). Biochemical analyses of recombinant proteins showed that MpCAPE has both AC and PDE activities. In Mp*CAPE*-promoter-*GUS* lines, GUS activity was specifically detected in the male sexual organ, the antheridium, suggesting Mp*CAPE* and thus cAMP signalling may be involved in the male reproductive process. *CAPE* orthologues are distributed only in basal land plants and charophytes that use motile sperm as the male gamete. CAPE is a subclass of class III AC and may be important in male organ and cell development in basal plants.

Cyclic AMP (cAMP) is a second messenger controlling many cellular functions and is synthesized by adenylyl cyclases (ACs). The intracellular concentration of cAMP is tightly regulated by the activities of AC and its degradation enzyme, cAMP phosphodiesterase (PDE)^1^,^2^.

Genes encoding adenylyl cyclases have been isolated from most of species of organisms and the physiological functions of cAMP have been well characterized^3^,^4^,^5^,^6^. For example, in *Escherichia coli*, cAMP binds to a receptor protein called the cAMP receptor protein (CRP) and the cAMP-CRP complex regulates the transcriptional activation of catabolite-sensitive operons^7^. In mammals, an increased cAMP level in response to the actions of hormones such as glucagon and adrenaline promotes phosphorylation of several intracellular enzymes via activation of protein kinase A, resulting in the enhancement of enzyme activities in glycogen and lipid metabolisms^8^,^9^. cAMP also plays roles in learning, memory and olfactory sensation by regulating gene expression and channel activity^10^,^11^. In mammals, ACs are classified into nine transmembrane enzymes (tmACs) and one soluble enzyme (sAC)^12^. The sAC appears evolutionarily distinct from tmACs and more closely related to cyanobacterial ACs, and plays central roles in sperm capacitation^13^,^14^,^15^.

Much effort has been put into isolating an AC from plants because of its crucial functions in other organisms^16^,^17^,^18^. There are three reports of the identification of *AC* genes in plants: *PSiP* in *Zea mays*^19^, *HpAC1* in *Hippeastrum x hybridum*^20^ and *AtKUP7* in *Arabidopsis thaliana*^21^. However, the amino acid sequence of PSiP did not show any homology to known ACs. Furthermore, *At*TTM3, the product of the *HpAC1* orthologue in *Arabidopsis thaliana*, is not an AC and has tripolyphosphatase activity^22^. Because the four classes of ACs identified show no sequence homology among them, indicating that they have emerged independently by convergent evolution^1^,^23^, it is possible that a new class of AC might exist in land plants. This may be why it has been difficult to identify an AC in plants, especially in angiosperms, despite the availability of huge amounts of genomic information. The genomic information on basal land plants is rapidly increasing through the progress of their genome projects^24^,^25^,^26^. Thus, now is a good time to search for *AC* genes in the genomes of basal land plants.

In this report, we searched for *AC* genes in the transcriptome data of the liverwort *Marchantia polymorpha* and found one sequence that had significant similarity to class III ACs (the universal class comprising ACs from bacteria to mammals). In addition to the AC domain in the C-terminal part, the N-terminal part showed similarity to cyclic nucleotide phosphodiesterases, so the gene was designated *COMBINED AC with PDE; CAPE*. MpCAPE has Mn^2+^-enhanced adenylyl cyclase activity and thus is the first functional class III AC from land plants. Moreover, promoter-*GUS* analysis showed Mp*CAPE* is specifically expressed in the antheridium. The distribution of *CAPE* orthologues corresponds to the streptophyte lineage that uses motile sperm as the male gamete for sexual reproduction. We discuss the involvement of CAPE in male reproductive organ development in streptophytes that have CAPE.

## Results

### Identification of an *AC* gene in *M. polymorpha*

A BLAST search was carried out against the transcriptome database of *M. polymorpha* to find genes encoding AC using the amino acid sequence of a cyanobacterial AC, CyaC^27^. We found one sequence showing significant similarity to the catalytic domain of CyaC. In addition to the AC domain, its deduced amino acid sequence contained a consensus sequence of the catalytic domain of PDEs, which are the degradation enzymes of the cyclic nucleotides, cAMP and cyclic GMP (cGMP). Thus, we designated the gene Mp*CAPE (COMBINED AC with PDE*) according to the gene nomenclature of *Marchantia*^28^. Mp*CAPE* is registered as Mapoly0068s0004 in the Phytozome web site (https://phytozome.jgi.doe.gov).

**Figure 1:**
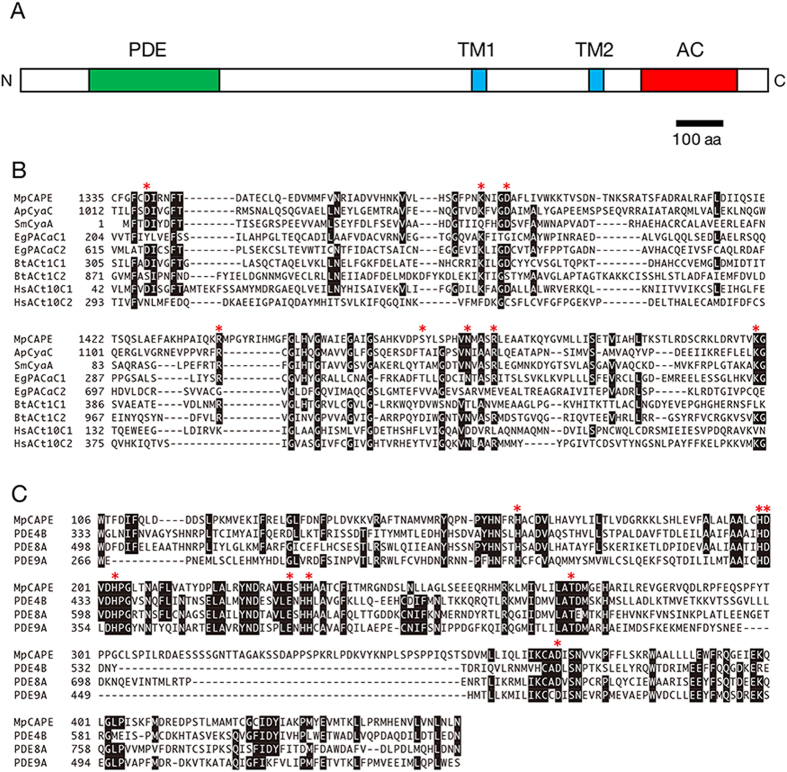
Domain organization and amino acid alignment of the AC and PDE domains of MpCAPE. (**A**) Schematic representation of the full-length MpCAPE. The catalytic domains of AC and PDE are shown as red and green boxes, respectively. Membrane-spanning regions are shown as blue boxes (TM1 and TM2). (**B**) An alignment of the AC domain of MpCAPE with *Arthrospira platensis* CyaC (ApCyaC), *Sinorhizobium meliloti* CyaA (SmCyaA), *Euglena gracilis* PACa (EgPACaC1 and EgPACaC2), *Bos taurus* type 1 AC (BtACt1C1 and BtACt2C2) and *Homo sapiens* type 10 AC (HsACt10C1 and HsACt10C2). Amino acids involved in binding the substrate ATP are indicated by red asterisks. (**C**) An alignment of the PDE domain of MpCAPE with *Homo sapiens* PDE4B, 8 A and 9 A. Amino acids involved in metal ion binding are indicated by red asterisks. Amino acid residues identical in majority of the sequences are shown in black.

Reverse transcription (RT)-PCR was performed using total RNA from *M. polymorpha* and the amino acid sequence of MpCAPE was determined (Supplementary Fig. S1). MpCAPE consisted of 1610 amino acids with a calculated molecular mass of 179 kDa. The C- and N-terminal parts showed similarity to the catalytic domains of class III ACs and PDEs, respectively (Fig. 1). Although the sequence identity was low, the consensus amino acids required for catalysis and substrate binding^29^,^30^ were well conserved in the AC domain of MpCAPE (Fig. 1B, asterisks). In PDEs, two metal ions, Zn^2+^ and Mg^2+^, are coordinated by highly conserved amino acids in the catalytic domain and indispensable for catalysis^31^,^32^. In the PDE domain of MpCAPE, all amino acids required for metal binding were conserved (Fig. 1C, red asterisks). In the middle part of MpCAPE, there were two hydrophobic segments, which were predicted to act as transmembrane helices (Fig. 1A and Supplementary Fig. S1).

### Complementation of the *AC*-deficient (*∆cya*) *E. coli* strain MK1010

**Figure 2:**
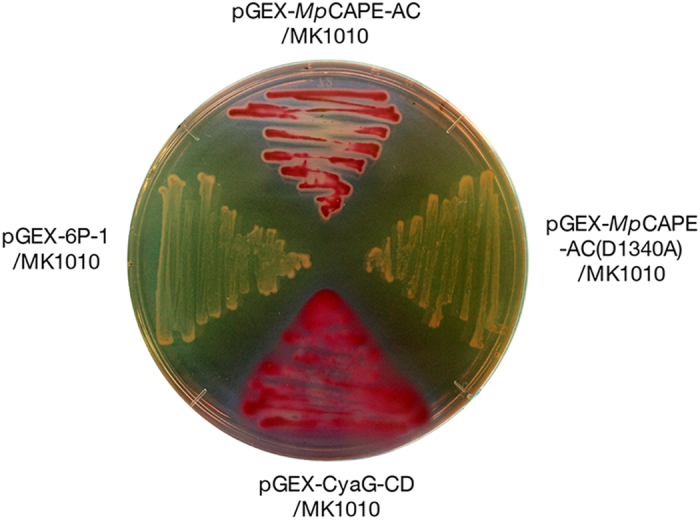
Functional complementation by Mp*CAPE-AC*. Colonies of the *E. coli* MK1010 (*∆cya*) strain transformed with pGEX-MpCAPE-AC (upper), pGEX-MpCAPE-AC(D1340A) (right), pGEX-6P-1 (left) or pGEX-CyaG-CD (lower) on MacConkey-maltose agar medium.

Wild-type *E. coli* cells utilize maltose in the presence of intracellular cAMP and form red colonies on MacConkey-maltose agar plates because of acid production during their growth, whereas *∆cya* mutants, including the *E. coli* MK1010 strain, form white colonies^33^. To examine whether MpCAPE has cAMP production activity, the expression vector pGEX-MpCAPE-AC, which contained the sequence of the putative AC domain of MpCAPE (1251–1610), was constructed and introduced into *E. coli* MK1010. The transformant pGEX-MpCAPE-AC/MK1010 formed red colonies (Fig. 2), similar to the transformant pGEX-CyaG-CD/MK1010, in which the catalytic domain of the cyanobacterial adenylyl cyclase CyaG was expressed^34^, whereas the transformant pGEX-6P-1/MK1010 (vector control) formed white colonies (Fig. 2). Thus, pGEX-MpCAPE-AC complemented the *AC*-deficient phenotype of *E. coli* MK1010. Additionally, pGEX-MpCAPE-AC(D1340A) was constructed because Asp-1340 corresponds to the metal binding site essential for the catalytic activity of mammalian AC^35^. The point mutation (D1340A) prevented pGEX-MpCAPE-AC from complementing the *AC* deficiency of *E. coli* MK1010 (Fig. 2). These results suggested that the AC domain of MpCAPE had an AC activity.

### Detection of cAMP in *E. coli* MK1010 harboring a gene for the AC of MpCAPE

The cellular cAMP levels in *E. coli* MK1010-based transformants were measured (Table 1). cAMP was not detected in *E. coli* MK1010, even when pGEX-6P-1 was introduced. In contrast, cAMP was detected in *E. coli* MK1010 transformed with pGEX-MpCAPE-AC, as was the case with the wild-type (*cya*^+^) strain *E. coli* DH5α. The D1340A mutation in MpCAPE-AC caused the loss of the ability to produce cAMP.

### *In vitro* AC activity of GST-MpCAPE-AC

To analyze its AC activity *in vitro*, the catalytic domain of MpCAPE-AC was produced as a GST fusion protein (GST-MpCAPE-AC) in *E. coli* and purified (Supplementary Fig. S2). The specific activity of GST-MpCAPE*-*AC with Mn^2+^ was 35-fold higher than that with Mg^2+^ (Table 2). The enhancement of the AC activity by Mn^2+^ is similar to other class III ACs^14^,^27^,^36^,^37^. The results of mutation analysis using GST-MpCAPE-AC(D1340A) showed that Asp-1340 was essential for AC activity (Table 2). Bicarbonate stimulates the activities of mammalian sAC and a cyanobacterial AC^15^. We examined the effect of bicarbonate on the AC activity of GST-MpCAPE-AC under the basal condition of its activity in the presence of Mg^2+^. The AC activity of GST-MpCAPE-AC was not affected by bicarbonate (Supplementary Table S1). The guanylyl cyclase (GC) activity of GST-MpCAPE-AC was tested by adding GTP instead of ATP to the enzyme assay mixtures but no GC activity was detected.

### *In vitro* PDE activity of His-MpCAPE-PDE

**Figure 3:**
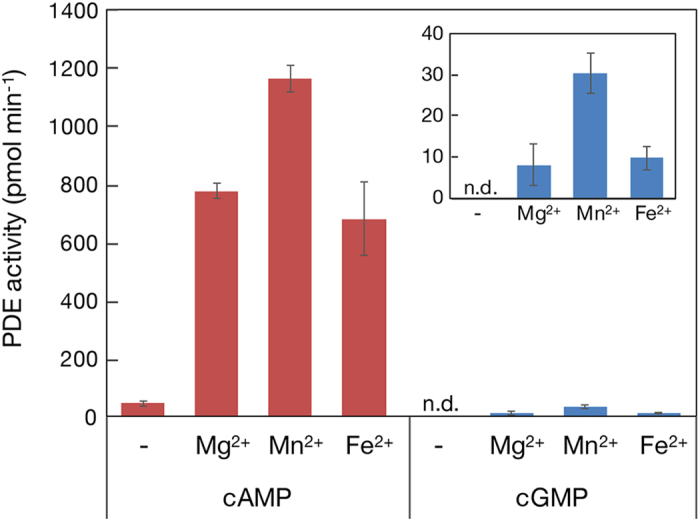
Effect of divalent cations on phosphodiesterase activity of His-MpCAPE-PDE. Phosphodiesterase activity of partially purified His-MpCAPE-PDE protein sample (13 μg) was measured in the presence or absence of various cations. cAMP or cGMP was used as substrate. The inset shows the magnification of the data with cGMP. Values indicate means ± SD (*n* = 3). n.d.: not detected.

To analyze its PDE activity *in vitro*, the catalytic domain of MpCAPE-PDE was produced as a 6 × His fusion protein (His-MpCAPE-PDE) in *E. coli* and purified using Ni^2+^-Sepharose column. The His-MpCAPE-PDE was eluted from the column with several other proteins (Supplementary Fig. S3A, lane 1). Using the partially purified protein sample, the PDE activity of His-MpCAPE-PDE was assayed in the presence or absence of divalent cations (Fig. 3). The result showed that His-MpCAPE-PDE hydrolyzed both cAMP and cGMP but cAMP was much more favorable substrate than cGMP. Divalent cations (Mg^2+^, Mn^2+^ and Fe^2+^) stimulated both cAMP- and cGMP-dependent activities. PDE activities in the presence of Mg^2+^ were examined using mutant proteins, His-MpCAPE-PDE-H199Q and His-MpCAPE-PDE-H203Q (Supplementary Fig. S3), in which the highly conserved histidines (Fig. 1C) were replaced by glutamine. PDE activities were completely disappeared by the mutations (Table S2). The result suggested that His-199 and His-203 were essential for the catalytic activity of MpCAPE-PDE and the activity detected in the partially purified sample was not due to the contamination proteins from *E. coli*.

### Tissue-specific expression pattern of Mp*CAPE*

**Figure 4:**
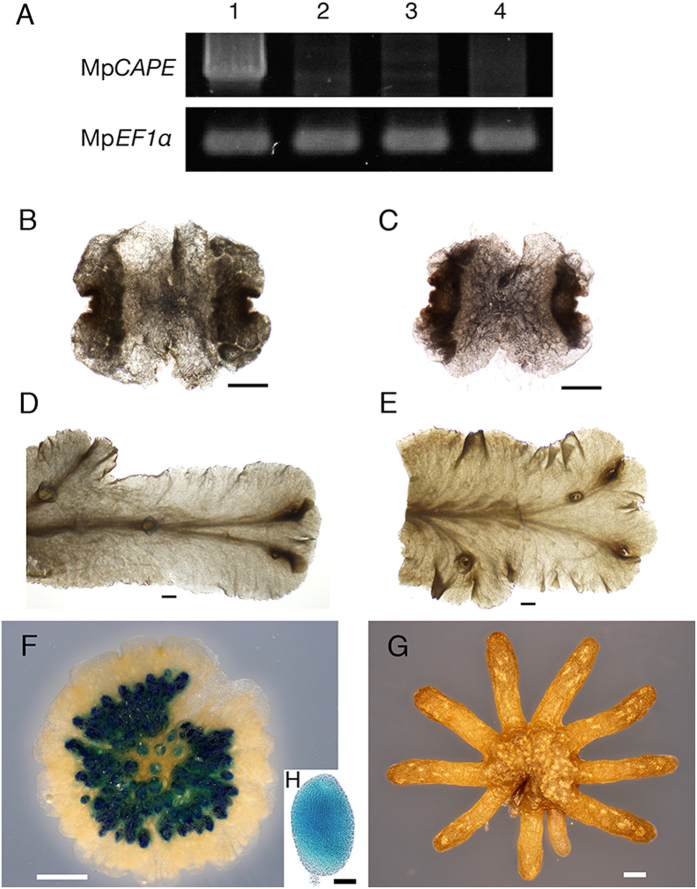
Mp*CAPE* mRNA accumulation in wild type and Mp*CAPE* promoter*-GUS* expression pattern in transgenic *M. polymorpha* plants. (**A**) RT-PCR analysis of *CAPE* mRNA accumulation in antheridiophores (lane 1), archegoniophores (lane 2), thalli from male accession Tak-1 (lane 3) and thalli from female accession Tak-2 (lane 4). *EF1*α encoding elongation factor 1α was used as a control. *GUS* expression in gemmalings (**B** and **C**), thalli (**D** and **E**), antheridiophore (**F**) and archegoniophore (**G**) of Mp*CAPE* promoter*-GUS* transgenic plants. Backgrounds of the transgenic plants were Tak-1 (**B**, **D** and **F**) and Tak-2 (**C**, **E** and **G**). GUS activity was detected only in the antheridiophore. Bars = 1 mm. (**H**) Antheridium dissected from a GUS-stained antheridiophore. Bar = 0.1 mm.

To examine the developmental and tissue-specific Mp*CAPE* expression, mRNA accumulation was examined by RT-PCR. The result showed that Mp*CAPE* specifically expressed in the antheridiophore, which is the male gametophore bearing the sexual organ, the antheridium (Fig. 4A). Next, we generated transgenic *M. polymorpha* lines expressing the *GUS* gene under the control of the Mp*CAPE* promoter. No *GUS* expression was detected in the vegetative growth phase (Fig. 4B–E). *GUS* expression was observed as dots in a sample of the antheridiophore (Fig. 4F). The GUS-stained dots looked like antheridia, so a *GUS*-stained antheridiophore was dissected and antheridia were prepared. *GUS* expression was observed in the antheridium (Fig. 4H). *GUS* expression was not detected in the female gametophore, the archegoniophore (Fig. 4G).

### *CAPE* orthologues in Streptophyta

**Figure 5:**
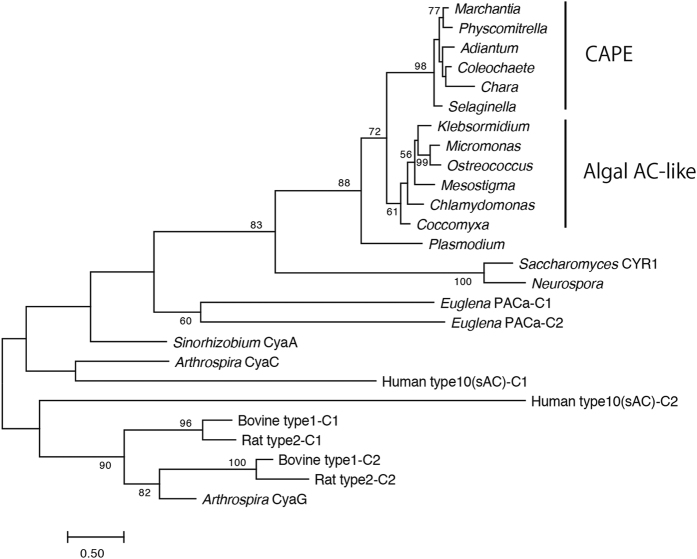
Phylogenetic tree of ACs. The phylogenetic tree was inferred using the maximum-likelihood method with the LG+Gamma model. Numbers represent support values (>50%) obtained with 100 bootstrap replicates using the MEGA7 software (LG+Gamma mode). The evolutionary distances were computed in units of the number of amino acid substitutions per site, as shown by the scale bar below the tree. The accession numbers of *AC* sequences used for phylogenetic analysis are shown in Supplementary Table S3.

We detected orthologous sequences in the moss *Physcomitrella patens* and the lycophyte *Selaginella moellendorffii* from their complete genome databases and in the charophyte *Coleochaete orbicularis* from its transcriptome data^38^ (Supplementary Fig. S4). Moreover, cDNA fragments of *CAPE* were amplified from the charophyte *Chara braunii* and the pteridophyte *Adiantum capillus-veneris* by RT-PCR, and their amino acid sequences were deduced (Supplementary Fig. S4). However, we could not find *CAPE* orthologues in gymnosperms (*Picea abies* and *Pinus taeda*) and angiosperms. Several homologous sequences that contained an AC domain but not a PDE domain were also found in green algae including the charophytes *Mesostigma viride* and *Klebsormidium flaccidum* from their transcriptome and complete genome databases^38^,^39^, respectively, and chlorophytes such as *Chlamydomonas reinhardtii, Coccomyxa subellipsoidea, Ostreococcus tauri* and *Micromonas pusilla*. Phylogenetic analysis showed that, regarding their AC domains, CAPEs and algal AC-like sequences were separated into two clades, with the branch supported by a high bootstrap value (72, Fig. 5).

## Discussion

We found a unique AC with a PDE domain, CAPE, in the genome of *M. polymorpha* and characterized the AC activity of MpCAPE using an *E. coli ∆cya* mutant and recombinant proteins. From the following results, we conclude that the protein encoded by Mp*CAPE* of *M. polymorpha* can produce cAMP and is the first functional class III AC identified from land plants. (i) The amino acid sequence deduced from the isolated cDNA exhibited similarity to the catalytic domain of class III ACs. Importantly, the amino acid residues required for the catalytic activity were conserved (Fig. 1). (ii) The cDNA fragment encoding the AC domain of Mp*CAPE* complemented *∆cya* of the *E. coli* MK1010 mutant strain (Fig. 2). (iii) cAMP was detected in the *E. coli* MK1010 strain transformed with the *AC* fragment of Mp*CAPE* (Table 1). (iv) A recombinant protein consisting of the AC domain of MpCAPE produced cAMP *in vitro* (Table 2).

The AC activity of MpCAPE was enhanced by Mn^2+^. Mn^2+^-enhancement has been observed in other ACs^14^,^27^,^36^,^37^ and is a common property in class III ACs. Thus, in the conservation of its amino acid sequence and its enzymatic properties, MpCAPE retains the characteristics of class III ACs. Furthermore, an aspartate residue, corresponding to Asp-1340 in MpCAPE, is essential for ATP binding by associating with Mg^2+^ in class III ACs^29^,^40^. To confirm that MpCAPE produces cAMP, an AC mutant in which Asp-1340 was replaced with Ala was tested for AC activities, i.e., complementation of the *E. coli ∆cya* mutant, cAMP production in *E. coli* cells and *in vitro* AC activity. In all experiments, the mutation (D1340A) resulted in the disappearance of AC activity, suggesting that Mp*CAPE* certainly encodes an AC.

The AC activities of MpCAPE with Mn^2+^ were 7 times higher than that of AtKUP7 of *Arabidopsis thaliana* (2.2 pmol min^−1^ mg^−1^ with Mn^2+^)^21^. Those proteins derived from land plants seem to have an equivalent AC activity. On the other hand, mammalian ACs show much higher activities, for example, 75 pmol min^−1^ mg^−1^ for a transmembrane AC (AC5) and 10 nmol min^−1^ mg^−1^ for soluble AC (sAC)^15^. It is possible that cAMP effectors might be localized in close proximity to ACs and relatively low AC activity might be enough for their activation. Also, there might be a mechanism to stimulate AC activity in plant cells.

MpCAPE is encoded by a single-copy gene and differs from the protein encoded by CUFF.20439 (Mapoly0178s0022) that was annotated as an *AC* gene in Higo *et al*.^41^. The Mapoly0178s0022 protein does not contain a PDE domain but does have a putative AC catalytic domain on its N-terminal side in which several conserved amino acids for catalysis are missing. Nevertheless, the enzymatic activity of the Mapoly0178s0022 protein needs to be characterized because it might still have the activity of an AC or GC, whose catalytic core sequence is homologous to that of AC^35^.

MpCAPE may be a membrane protein and has two-membrane-spanning helices between the AC and PDE domains, so both domains may face on the same side of a membrane, likely the cytosolic space because of the presence of ATP pool as substrate for the AC activity. The cellular level of cAMP could be tightly controlled by MpCAPE through synthesis and hydrolysis. It is thought that, because there are different cAMP effectors that regulate each specific signalling process in a cell, cAMP must be prevented from free diffusion and localized in restricted areas to activate specific signalling pathways^42^. PDEs have a critical role in the compartmentalization of cAMP signalling^43^,^44^. MpCAPE allows for such spatial regulation of cAMP by itself.

The gametophyte of *M. polymorpha* is dioecious. When entering the sexual reproductive phase, the male and female gametophytes develop individual sexual organs, the antheridium and archegonium, on special gametophores called the antheridiophore and archegoniophore, respectively^45^. It has been shown that a number of specific genes are expressed in the antheridium of *M. polymorpha*^41^. In our Mp*CAPE* promoter-*GUS* experiment, the promoter activity was specifically detected in the antheridium (Fig. 4). Motile sperm cells with two flagella are developed in the antheridium of *M. polymorpha* 45. As far as we know, there have been no reports showing a role for cAMP in antheridium formation or spermatogenesis in *M. polymorpha*. However, in animal cells including mammalian cells, the function of cAMP in spermatogenesis has been characterized and it has been shown to be an indispensable factor in sperm physiology, such as the regulation of sperm capacitation, the acrosome reaction and the activation of sperm motility^13^,^46^,^47^,^48^,^49^. In addition to the physiological roles of cAMP, the ACs that play roles in spermatogenesis have been analyzed^13^,^50^. Mammalian ACs include nine transmembrane AC isoforms (tmAC1–9) and one soluble AC (sAC)^51^. The sAC not only has a different topology to tmACs, but also a distinct catalytic domain, which is more closely related to cyanobacterial adenylyl cyclases^14^,^15^. In mammalian sperms, the sAC is the main source of cAMP and has a dominant role in the acquisition of fertilizing capacity^13^. The fact that AC has a dominant function in sperm physiology and has been genetically inherited during the evolution of mammals may suggest a possible role for cAMP in the reproductive organ development of *M. polymorpha*. A recent paper has shown the expression of a cAMP-dependent protein kinase and cyclic nucleotide-gated ion channel in the antheridium of *M. polymorpha*^41^. It is likely that these proteins function as signalling factors downstream of MpCAPE.

The functional importance of the MpCAPE in the male organ also seems to be supported by the distribution of CAPEs in Streptophyta; charophytes plus land plants. In charophytes, *CAPE* genes were identified in *Chara* and *Coleochaete*, but not in the genome of *Klebsormidium*, although an algal *AC*-like sequence is present in its genome. According to the phylogeny of the charophyte lineage^52^,^53^, since Charales appeared and diverged after the establishment of Mesostigmatales and Klebsormidiales, CAPE must have appeared during the evolutionary process between Klebsormidiales and Charales. Charales first developed a motile sperm with flagella in Streptophyta and the architecture of the mature sperm is remarkably similar to that in basal land plants^54^. The occurrence of CAPE is in consistent with the emergence of motile sperm as the male gamete in Charales. Furthermore, because the AC domains of CAPEs were sister to the algal AC-like sequences of *Mesostigma, Klebsormidium* and chlorophytes in the phylogenetic analysis of class III ACs (Fig. 5), we can infer that CAPEs arose from the fusion of a PDE domain with an algal AC-like sequence.

Zygnematophyceae, a class of charophytes, have been proposed as the sister group of land plants, but do not produce motile sperm cells and alternatively use conjugation system for sexual reproduction^52^,^53^. We could not find homologous sequences of CAPE in the transcriptome data of *Spirogyra pratensis*^38^ and in the GenBank sequence data derived from the members of Zygnematophyceae. Complete genome sequence data from Zygnematophyceae will reveal the presence or absence of CAPE in this lineage.

In land plants, CAPEs are present in *Marchantia, Physcomitrella, Selaginella* and *Adiantum*, which all use motile sperm as male gametes^54^. However, neither CAPEs nor class III ACs including algal AC-like sequences are present in gymnosperms (*Picea abies* and *Pinus taeda*) and angiosperms that use a non-motile sperm cell delivered to the egg cell. CAPE seems to have been lost during the evolutionary process from ferns to seed plants. The disappearance of CAPEs in land plant lineages also corresponds with the loss of motile sperm. In summary, the distribution of CAPEs coincides well with the use of motile sperm in Streptophyta. It will be interesting to investigate whether CAPEs exist in the gymnosperms *Ginkgo* and *Cycas*, which are unique in having motile sperms as male gametes.

Using recently developed molecular techniques for *M. polymorpha*^55^, analysis of Mp*CAPE* mutants constructed by gene targeting should clarify the physiological roles of cAMP in the male organ of *M. polymorpha*. Information on the physiological and molecular functions of Mp*CAPE* will contribute to our understanding of the role of cAMP in other plants.

## Methods

### Culture and growth conditions of *Marchantia polymorpha*

Male and female accessions of *M. polymorpha*, Takaragaike (Tak)-1 and Tak-2, respectively, were cultured aseptically at 22 °C on 1/2-strength Gamborg’s B5 agar medium under continuous white light conditions, or grown on vermiculite soaked with 1/1000-diluted Hyponex solution (HYPONex JAPAN Co. Ltd, Osaka, Japan) at 22 °C under 16 h white fluorescent light supplemented with far-red LED light and 8 h dark conditions.

### Cloning of genes encoding an AC from *M. polymorpha*

A Mp*CAPE* cDNA was obtained by RT-PCR using total RNA prepared from antheridiophores of *M. polymorpha* Tak-1 and the primers MpCAPE-f (5′-CACCATGCATGCTTGCTTTGAGGG-3′) and MpCAPE-r (5′-CTACTTCTCGGTGAGTTCTC-3′). The amplified DNA fragment (approximately 5 kb) was excised from an agarose gel and purified using a NucleoSpin Extract kit (Macherey-Nagel, Germany). The purified DNA fragment was cloned into the cloning vector pENTR/D-TOPO (Thermo Fisher Scientific, USA). The nucleotide sequence was determined using a DNA sequencer (Genetic Analyzer 3130, Thermo Fisher Scientific, USA).

### Construction of the expression plasmid for the GST-MpCAPE-AC protein

A DNA fragment encoding the AC domain of MpCAPE (1251–1610) was amplified by PCR using the primers MpCAPE-EX-f (5′-GGGATCCCCGGAATTCCAGCCTATTGAGCGCATGGT-3′) and MpCAPE-EX-r (5′-AGTCACGATGCGGCCGCCTACTTCTCGGTGAGTTCTC-3′), and cDNA as a template. The amplified DNA was cloned into the *Eco*RI-*Not*I site of the pGEX-6P-1 vector with an In-fusion Cloning kit (Takara, Japan). The resulting plasmid was named pGEX-MpCAPE-AC.

The GST-MpCAPE-AC(D1340A) mutant was constructed with mismatched oligonucleotides and PCR. PCRs were performed with the primers MpCAPE-EX-f and MpAC-D2A-r (5′-AGTTTCGTATGgCACAGAATCCA-3′) or MpAC-D2A-f (5′-TGGATTCTGTGcCATACGAAACT-3′) and MpCAPE-EX-r. The resulting amplified DNA fragments were mixed and PCR was repeated with the primers MpCAPE-EX-f and MpCAPE-EX-r using the DNA mixture as a template. The amplified DNA fragment was cloned into the *Eco*RI-*Not*I site of the pGEX-6P-1 vector as described above. The resulting plasmid was named pGEX-MpCAPE-AC(D1340A).

### Expression and purification of GST-MpCAPE-AC proteins

The constructed expression plasmids were introduced into an *E. coli AC* mutant, MK1010^33^, to express the AC domain of MpCAPE as a fusion protein with an affinity tag, glutathione-S-transferase (GST). The transformants were grown at 25 °C in LB medium (1.5 L) containing ampicillin (100 μg ml^−1^) and kanamycin (50 μg ml^−1^). Protein expression was induced by adding 0.1 mM isopropyl-ß-D-thiogalactopyranoside (IPTG) at OD_600_ = 0.5. The cells were grown at 25 °C for 24 h, harvested by centrifugation, resuspended in 20 mL of TEG buffer (50 mM Tris-HCl (pH 8.0), 1 mM EDTA, 10% (w/v) glycerol, 0.5 M NaCl) and disrupted by sonication. The cell extracts were centrifuged at 15,000 ×  *g* for 30 min and the supernatants were loaded onto a 1 mL glutathione column (GSTrap HP, GE Healthcare, USA). The columns were washed with TEG buffer and the proteins were eluted with 5 mM glutathione in TEG buffer.

### Adenylyl cyclase activity assay

*In vitro* adenylyl cyclase reactions (9 μg of protein) were performed in 0.1 mL assay buffer containing 50 mM Tris-HCl (pH 7.5), 1 mM ATP, 1 mM DTT, and 1 mM MgCl_2_ or MnCl_2_ at 37 °C for 30 min. The enzyme reaction was terminated by adding 1 mL of 5% (v/v) trichloroacetic acid (TCA). After removing the TCA from each reaction mixture by extracting with ethyl ether, the samples were lyophilized. cAMP contents were measured with an enzyme immuno assay system (cAMP EIA system, GE Healthcare, USA) according to the manufacturer’s instructions.

### Complementation test of the adenylyl cyclase deficiency of *E. coli* MK1010

The transformants (MK1010 cells harboring the constructed vectors described above) were streaked onto MacConkey agar plates (Difco, Germany) containing 1% (w/v) maltose, 100 μg ml^−1^ ampicillin and 50 μg ml^−1^ kanamycin and grown at 25 °C.

### Construction of the expression plasmids for His-MpCAPE-PDE proteins

A DNA fragment encoding the PDE domain of MpCAPE (101–479) was amplified by PCR using the primers MpCAPE-PDE-EX-f (5′-TCGCGGATCCGAATTCCAGGGAATTAACTCGTGGAC-3′) and MpCAPE-PDE-EX-r (5′-GGTGGTGGTGCTCGAGTTAAAAGGGACCAAGAATCTGCT-3′), and cDNA as a template. The amplified DNA was cloned into the *Eco*RI-*Xho*I site of the pET28a vector with an In-fusion Cloning kit (Takara, Japan). The resulting plasmid was named pET-MpCAPE-PDE.

The His-MpCAPE-PDE(H199Q) and –PDE(H203Q) mutants were constructed with mismatched oligonucleotides and PCR in the same manner as described above for the construction of GST-MpCAPE-AC(D1340A) mutant. The amplified DNA fragments were cloned into the *Eco*RI-*Xho*I site of the pET28a vector. The resulting plasmids were named pET-MpCAPE-PDE(H199Q) and pET-MpCAPE-PDE(H203Q).

### Expression and purification of His-MpCAPE-PDE proteins

The constructed expression plasmids were introduced into an *E. coli* Rosetta2(DE3)pLysS strain, to express the PDE domain of MpCAPE as a fusion protein with an affinity tag (6 ×His) and an epitope tag (T7-tag). The transformants were grown at 25 °C in LB medium (4 L) containing kanamycin (50 μg ml^−1^) and chloramphenicol (30 μg ml^−1^). Protein expression was induced by adding 0.1 mM IPTG at OD_600_ = 0.35. The cells were grown at 25 °C for 7 h, harvested by centrifugation, resuspended in 70 mL of TNG buffer (50 mM Tris-HCl (pH 8.0), 0.2 M NaCl, 10% (w/v) glycerol) and disrupted by sonication. The cell extracts were centrifuged at 15,000 × *g* for 30 min and the supernatants were loaded onto a 1 mL Ni^2+^ column (HisTrap HP, GE Healthcare, USA). The columns were washed with TNG buffer containing 100 mM imidazole and the proteins were eluted with 200 mM imidazole in TNG buffer.

### Phosphodiesterase activity assay

*In vitro* phosphodiesterase reactions (13 μg of partially purified protein) were performed in 0.1 mL assay buffer containing 30 mM Tris-HCl (pH 8.0), 0.5 mM cAMP or cGMP, 0.1% (v/v) 2-mercaptoethanol, and 0.5 mM MgCl_2_, MnCl_2_ or FeCl_2_ at 37 °C for 20 min. The enzyme reaction was terminated by incubation at 100 °C for 10 min. After centrifugation at 15,000× *g* for 10 min, the supernatants were analyzed by reverse-phase column chromatography (COSMOSIL 5C_18_-AR; 4.6 × 250 mm; Nacalai, Kyoto, Japan) using 30 mM sodium phosphate buffer (pH 5.0) containing 5%(v/v) acetonitrile as the mobile phase at a flow rate of 1.0 ml min^−1^. The effluent was monitored at 259 nm to detect AMP and GMP hydrolyzed from cAMP and cGMP by phosphodiesterase, respectively.

### *Promoter-GUS* construct and GUS staining

An 11-kb genomic DNA fragment, upstream of the triplet encoding His-259 of MpCAPE, was amplified by PCR using the primers PMpCAPE-f (5′-CACCACCAGCTAGGGAAACAGGGT-3′) and PMpCAPE-r (5′-GTGTCTCTGCTCCTCTTCAC-3′) and genomic DNA of *M. polymorpha* Tak-1 as a template. The amplified DNA fragment was cloned into the cloning vector pENTR/D-TOPO (Thermo Fisher Scientific, USA). The resulting plasmid was used for LR recombination by the Gateway technique (Thermo Fisher Scientific, USA) with pMpGWB104^56^ containing a *GUS* gene to produce the binary vector pMpGWB-Pcape. The GUS protein should be expressed as a translational fusion with the N-terminal fragment of MpCAPE (Met-1 to His-259). *Agrobacterium*-mediated transformation was carried out using regenerating thalli of *M. polymorpha* Tak-1 and Tak-2^57^. Hygromycin-resistant plantlets were selected to establish isogenic lines. Gemmae obtained from the isogenic lines were planted on vermiculite, grown for an appropriate length of time and histochemically stained to detect GUS activity.

### Sequence determination of Mp*CAPE* orthologues of *C. braunii* and *A. capillus-veneris*

Primer pairs, CbCAPE-F (5′-CACCACGGTCCTCGTCTCAGTGTT-3′) & CbCAPE-R (5′-CGTCGTGTGCAACCCTATTT-3′) and AcCAPE-F (5′-CACCCCAAAGATGGTGGAACGAAT-3′) & AcCAPE-R (5′-TGGCATACCAAAATTCCACA-3′) for amplification of *C. braunii* and *A. capillus-veneris CAPE* cDNAs, respectively, were designed from the RNA-seq data obtained with an Illumina Hiseq2000. RT-PCR was performed using total RNA prepared from thalli of *C. braunii* or prothallia of *A. capillus-veneris* and the primers noted above. The amplified DNA fragments were cloned into the cloning vector pENTR/D-TOPO (Thermo Fisher Scientific, USA). The nucleotide sequences were determined using a DNA sequencer (Genetic Analyzer 3130, Thermo Fisher Scientific, USA).

### Phylogenetic analysis

The amino acid sequences of the catalytic domains of adenylyl cyclases (ACs) (Table S3) were aligned using the ClustalX 2.0 program^58^. After removing ambiguously aligned regions, phylogenetic analysis was performed with a data matrix consisting of 134 amino acids for the AC domains from 26 operational taxonomic units (OTUs). A maximum-likelihood (ML) tree for the AC domains was determined using the MEGA7 software^59^, based on the LG^60^ + Gamma model. Bootstrap analysis of the ML tree was performed with 100 replications.

### Other analytical procedures

Protein content was measured with a Bio-Rad protein assay kit using gamma-globulin as the standard.

## Additional Information

**How to cite this article**: Kasahara, M. *et al*. An adenylyl cyclase with a phosphodiesterase domain in basal plants with a motile sperm system. *Sci. Rep.* **6**, 39232; doi: 10.1038/srep39232 (2016).

**Publisher's note:** Springer Nature remains neutral with regard to jurisdictional claims in published maps and institutional affiliations.

## Supplementary Material

Supplementary Information

## Figures and Tables

**Table 1:** cAMP levels in *E. coli* strains.

*E. coli* strains	cAMP level (pmol mg^−1^ protein)^a^
DH5α (*cya*^+^)	19.5 ± 3.3
MK1010 (*∆cya*)	n.d.^b^
pGEX-6P-1/MK1010	n.d.^b^
pGEX-MpCAPE-AC/MK1010	2.38 ± 0.06
pGEX-MpCAPE-AC(D1340A)/MK1010	n.d.^b^

^a^Mean values ± standard deviation (*n* = 3).

^b^n.d. = not detected.

**Table 2:** Adenylyl cyclase activity of GST-MpCAPE-AC.

Proteins	Specific activity (pmol min^−1^ mg^−1^)^a^
GST-MpCAPE-AC (Mg^2+^)	0.44 ± 0.09
GST-MpCAPE-AC (Mn^2+^)	15.5 ± 1.2
GST-MpCAPE-AC(D1340A) (Mg^2+^)	n.d.^b^
GST-MpCAPE-AC(D1340A) (Mn^2+^)	n.d.^b^
GST (Mg^2+^)	n.d.^b^
GST (Mn^2+^)	n.d.^b^

^a^Mean values ± standard deviation (*n* = 4).

^b^n.d. = not detected.
