# Supplementary material for: An adenylyl cyclase with a phosphodiesterase domain in basal plants with a motile sperm system
Source: Sci Rep. 2016 Dec 16;6:39232. doi: 10.1038/srep39232 (PMC5159850; doi:10.1038/srep39232)
Supplement: Supplementary Information [file srep39232-s1.pdf]

Supplementary Information for

An adenylyl cyclase with a phosphodiesterase domain in basal plants with a motile sperm system

Masahiro Kasahara*, Noriyuki Suetsugu, Yuki Urano, Chiaki Yamamoto, Mikiya Ohmori, Yuki Takada, Shujiro Okuda, Tomoaki Nishiyama, Hidetoshi Sakayama, Takayuki Kohchi and Fumio Takahashi

*To whom correspondence should be addressed: kasa@sk.ritsumei.ac.jp

This contains Supplementary Tables S1-S3 and Figure S1-S4.

Table S1 Effect of bicarbonate on adenylyl cyclase activity of GST-MpCAPE-AC

Proteins	Specific activity (pmol min^{-1} mg^{-1}) [a,b]
GST-MpCAPE-AC (Mg^{2+})	0.39 ± 0.05
GST-MpCAPE-AC (Mg^{2+}, 50 mM HCO_3^-)	0.35 ± 0.04

[a]Mean values ± standard deviation (n = 4)

[b]Adenylyl cyclase activities were assayed as described in Methods except for using 200 mM Tris-HCl (pH 7.5) instead of 50 mM Tris-HCl (pH 7.5). One mM $MgCl_2$ and 50 mM $NaHCO_3$ were used.

Table S2 cAMP phosphodiesterase activity of His-MpCAPE-PDE proteins

Proteins	Phosphodiesterase activity (pmol min^{-1}) [a,b]
His-MpCAPE-PDE	573 ± 19
His-MpCAPE-PDE-H199Q	n.d.[c]
His-MpCAPE-PDE-H203Q	n.d.[c]

[a]Mean values ± standard deviation (n = 3)

[b]cAMP phosphodiesterase activities were assayed as described in Methods with $MgCl_2$.

[c]n.d. = not detected

Table S3 Accession number of registered adenylyl cyclase genes in phylogenetic tree

Species	Abbreviation	Accession No.
Viridiplantae and Euglena		
Marchantia polymorpha	*Marchantia*	Mapoly0068s0004[*1]
Physcomitrella patens	*Physcomitrella*	Pp3c8_21740[*1]
Selaginella moellendorffii	*Selaginella*	427736[*1, 2]
Adiantum capillus-veneris	*Adiantum*	LC164752
Coleochaete orbicularis	*Coleochaete*	GBSL01008739.1[*3]
Chara braunii	*Chara*	LC169160
Klebsormidium flaccidum	*Klebsormidium*	kfl00481_0040[*4]
Mesostigma viride	*Mesostigma*	GBSK01038777.1[*5]
Chlamydomonas reinhardtii	*Chlamydomonas*	EDP05013
Ostreococcus tauri	*Ostreococcus*	XM_003079447
Coccomyxa subellipsoidea	*Coccomyxa*	XM_005649187
Micromonas pusilla	*Micromonas*	XM_003056838
Euglena gracilis	*Euglena* PACa-C1	AM181334
	Euglena PACa-C2	AM181334
Alveolata		
Plasmodium falciparum	*Plasmodium*	AJ289136
Fungi		
Saccharomyces cerevisiae	*Saccharomyces* CYR1	M12057
Neurospora crassa	*Neurospora*	XM_011394894
Bacteria and Cyanobacteria		
Sinorhizobium meliloti	*Shinorhizobium* CyaA	M35096
Arthrospira platensis	*Arthrospira* CyaC	D49692
	Arthrospira CyaG	D49531
Mammalia		
Rattus norvegicus	Rat type2-C1	AAA40682
	Rat type2-C2	AAA40682
Bos taurus	Bovine type1-C1	NP_776654
	Bovine type1-C2	NP_776654
Homo sapiens	Human type10(sAC)-C1	NP_060887
	Human type10(sAC)-C2	NP_060887

[*1]https://phytozome.jgi.doe.gov

[*2]The cDNA sequence in this study was reconstituted from the predicted ORF sequence (427736) and the genome DNA sequence to obtain the best match to the sequence from *Marchantia polymorpha*.

[*3]NCBI TSA accession number GBSL01000000 (Ju et al. *Nat. Plants*, **1**, 14004 (2015))

[*4]http://www.plantmorphogenesis.bio.titech.ac.jp/~algae_genome_project/klebsormidium/

[*5]NCBI TSA accession number GBSK01000000 (Ju et al. *Nat. Plants*, **1**, 14004 (2015))

```
   1 MEKPELKDDKGKDQEENDEAKNKRPRDLRIPLLNLERAREYVPPASPTEK
  51 TASDSEMLIISELPNIGQPDFSFSPVLEREDSPKLKAPSALSDEAVQQVL
 101 QGINSWTFDIFQLDDDSLPKMVEKIFRELGLFDNFPLDVKKVRAFTNAMV
 151 MRYQPNPYHNFRHACDVLHAVYLILTLVDGRKKLSHLEVFALALAALCHD
 201 VDHPGLTNAFLVATYDPLALRYNDRAVLESHHAATCFITMRGNDSLNLLA
 251 GLSEEEQRHMRKLMIVLILATDMGEHARILREVGERVQDLRPFEQSPFYT
 301 PPGCLSPILRDAESSSSGNTTAGAKSSDAPPSPKRLPDKVYKNPLSPSPP
 351 IQSTSDVMLLIQLIIKCADISNVVKPFFLSKRWAALLLLEWFRQGEIEKQ
 401 LGLPISKFMDREDPSTLMAMTCGCIDYIAKPMYEVMTKLLPRMHENVLVN
 451 LNLNRQMWSTFSTNGRRASETAQQILGPFAPPPIPKGEAVQEGYQQDHSK
 501 LEGKLVATFSGQYVSSDVPSRKLESSPSAKSLGSVSEDSEETVENVEDSP
 551 ILSVTDRSSEFSRLGSEAGSSVKTSPQFLSRAGTEAPSSPQSPLQASQEQ
 601 RGSPSPGVQYRLSPRGPGSPSVPVTQISGNPESPGYRETYGPGGSQPAAM
 651 EVPFVQKSPHEPVAVDVDVAASQQSADVIAPLSKPQALQVKQPVVPTVSR
 701 GVHFDDSELTASERSELSSSIQSGSSPDLALRVSFVAINPDSDSVKVLIE
 751 QSDPLSTATIEQRDPDSPRMTGLEPFLEEVAASSSESKAEPETEDEYNQR
 801 YEAPLSLLFGAPTREELMLPAKIGKDKNASLVSALLPGANNLVNDRKQNA
 851 WEQMAFRSPSEQAASRGSILTTVPSFANSDGDLEKSSSLRKGPPPGFWEA
 901 LRTHPRVNRLNRALESKTWNALLIVATLVALFADDVVKGFLPKHADVYES
 951 HILTACLSLFLAESALLCIFRDRYFFSFFFWLDMLGSVSLVPLVIGITAQ
1001 NLVIARTGRAAKTVTRFSKTLQASHIQQQIVHHIPVLRVFKFFGFKRDST
1051 LESPDYEEEEKFLSKPSQLWSRLAELTSQKLILGVLIIMILTPYFNNSEK
1101 DLAPLVSLDPLDDYLIGSPNFNLTVERVINMTKRHGYNLLYLGVKASCRS
1151 IKEGGYSYCLGIDVAGVEKYQQILPNVDEEPDGRQAAQEEFRPTELISVT
1201 SDSSRAQAYYSIKKKSRFKYGMNIAMTVLILLLLAAWCFFLSRDSNRLLI
1251 QPIERMVQFVKELAEDPVSFAGKTVVKPTGDSSKIMETFYVEAALVKIAS
1301 LTKVALGDAGMDILSVNLKGSEFNPMLPGKKIRACFGFCDIRNFTDATEC
1351 LQEDVMMFVNRIADVVHNKVVLHSGFPNKNIGDAFLIVWKKTVSDNTNKS
1401 RATSFADRALRAFLDIIQSIETSQSLAEFAKHPAIQKRMPGYRIHMGFGL
1451 HVGWAIEGAIGSAHKVDPSYLSPHVNMASRLEAATKQYGVMLLISETVIA
1501 HLTKSTLRDSCRKLDRVTVKGSQDPMVLYTFDIPLFQQDLRGNPQEYRDI
1551 FEEAVDSYIDGDWDIALERLQECQTLWPTDKPATVLLTFMASHNNIAPEN
1601 WAGFRELTEK
```

Figure S1. Amino acid sequence of MpCAPE. The catalytic domains of adenylyl cyclase (AC) and phosphodiesterase (PDE) are indicated by red (1335-1521) and green (106-452), respectively. Two membrane-spanning regions (TM1: 974-997 and TM2: 1220-1242) are indicated by blue. The membrane-spanning regions were predicted by a web site, TMHMM (http://www.cbs.dtu.dk/services/TMHMM/).

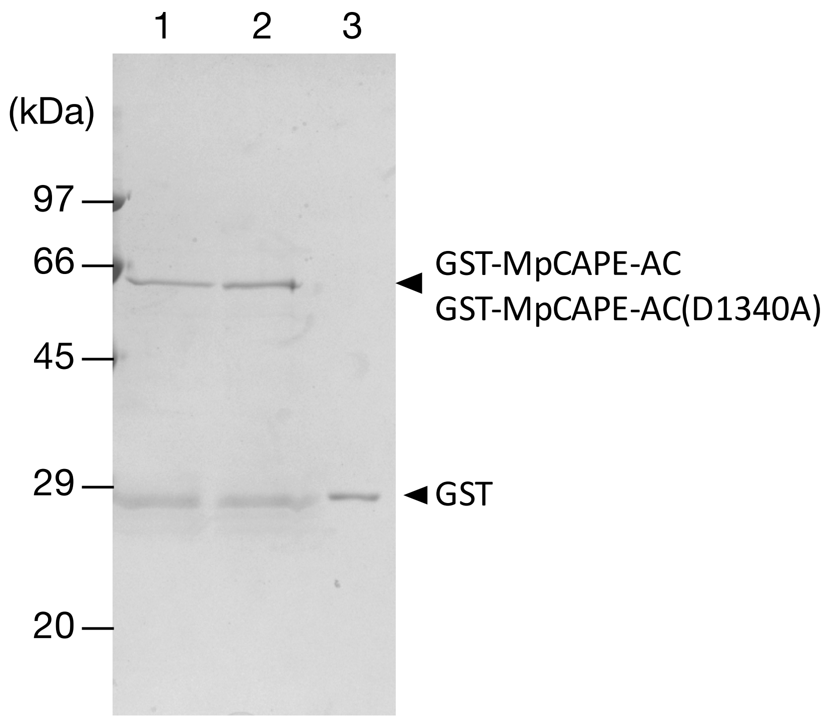

Figure S2. Purification of GST-MpCAPE-AC protein. Each affinity-purified protein (1.5 µg) was loaded onto a 12% SDS-PAGE gel for electrophoresis. The gel was stained with Coomassie Brilliant Blue. Lane1, GST-MpCAPE-AC; lane 2, GST-MpCAPE-AC(D1340A); lane 3, GST. The positions of size markers are shown at the left side of the gel.

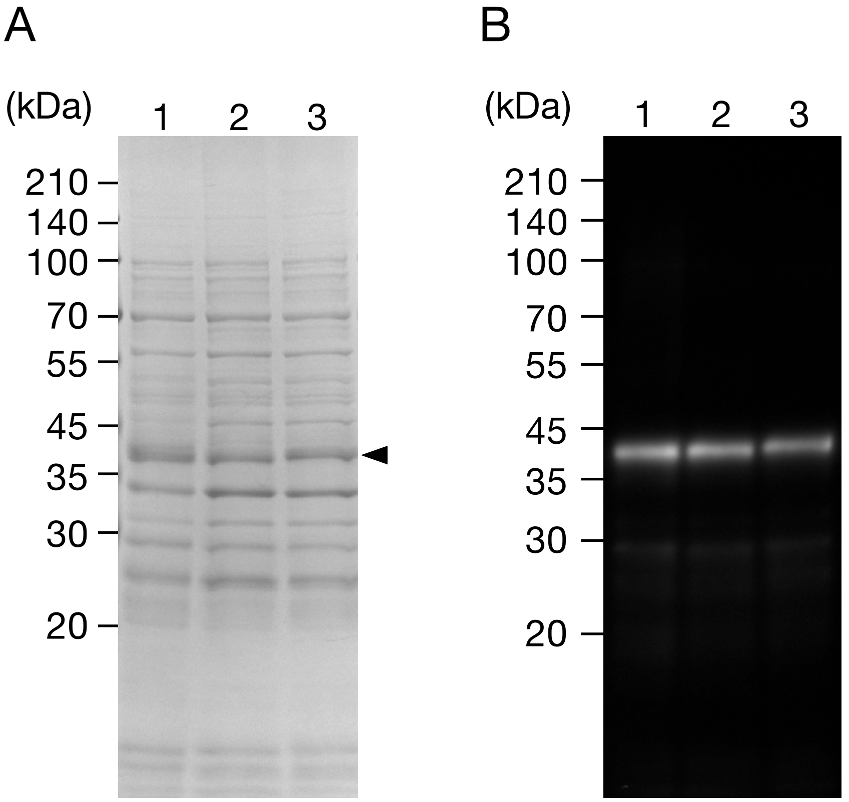

Figure S3. Partial purification and immunodetection of His-MpCAPE-PDE proteins. (A) Partially purified protein samples (10 µg) were loaded onto a 12% SDS-PAGE gel for electrophoresis. The gel was stained with Coomassie Brilliant Blue. The arrow head indicates the position of the His-MpCAPE-PDE proteins. The positions of size markers are shown at the left side of the gel. Lane 1: His-MpCAPE-PDE, lane 2: His-MpCAPE-PDE-H199Q, lane 3: His-MpCAPE-PDE-H203Q. (B) The partially purified protein samples (0.1 µg) were loaded onto a 12% SDS-PAGE gel for electrophoresis and subjected to immunoblotting with an anti-T7 epitope tag antibody for detection of His-MpCAPE-PDE proteins. The positions of size markers are shown at the left side of the gel. Lane 1: His-MpCAPE-PDE, lane 2: His-MpCAPE-PDE-H199Q, lane 3: His-MpCAPE-PDE-H203Q.

```
MpCAPE     1 M-------------EKPELKDDKGKDQEENDEAKNKRPRDLRIPLLN----------LERAREYVPPASPTEKTASDSEMLIISELPNIGQPDFSFSPVL     77
PpCAPE     1 M-------------------------------------------------------------------SEQENSAPKSGTFQVSEGE-------------     20
SmCAPE     1 M---------------------------------------------------------EHKRPSIIRISKPRHSLLIQEEVLAAKVDVVGR---------     34
CbCAPE     1 MSRYNASAGSWSRMVANPLSKTSPRGGDTDEVGENPKPQRSQTSGPQDMADWSAKGGLGHLGGSGKGLGKVSGKDADDDELERAPPFESPRSHDAESRAA    100
CoCAPE     1 M-------------------------------------------------------------SKTPELGRALTSSVKASAALLKEAHDLQG---------     30

MpCAPE    78 EREDSPKLKAPSALSDEAVQQVLQGINSWTFDIFQLDDDSLPKMVEKIFRELGLFDNFPLDVKKVRAFTN--AMVMRYQPN-PYHNFRHACDVLHAVYLI    174
PpCAPE    21 -------------------HKSLQGIESWSFDVFQVEDDQLPMLVEKIFRSLNIFNNFPIDMHKFRAFVR--AMVARYQPN-PYHCFRHACDVLHAVYLI     98
SmCAPE    35 ----------------HELEGLWQELDSWSFDIFKVATEDLPKMAERIFVNLGLLTTLPLDVQKLKAFIECVGMMAQYRAHVPFHNFRHACDVLHAMYLF    118
AcCAPE     1                                          PKMVERIFHDIGLFEALPLDASKFFGFVN--SIMGQYRAHVPYHNFRHACDVLHATYLL     57
CbCAPE   101 VIQAFLEEQNVIYDTSKSISELLEDIDSWEFDIFLVKEEDLNRVVEHVCERLGLLDTFGIDRNAFTIFVS--HIQSQYQSN-AYHNFRHACDVMHCTYIF    197
CoCAPE    31 ---------GIPLLTEEDIEVVLQDIDGLSFDIFRIREEDLHKLVERTFQRLGLFEEFPISRIVFRAFVH--AIQEQYQPN-TYHNFRHACDVVHAMYLM    118

MpCAPE   175 LTLVDGRKKLSHLEVFALALAALCHDVDHPGLTNAFLVATYDPLALRYNDRAVLESHHAATCFITMRGNDSLNLLAGLSEEEQRHMRKLMIVLILATDMG    274
PpCAPE    99 LTLAEGAQRLNQMEIFAVALAAVCHDVDHPGLTNAFLVAASDPISLRYNDKAVLESHHAATAFLTMRGHDNVNIMFSLDEPHQRHLRRLVLSCILATDMG    198
SmCAPE   119 LKLGGADEKFNELEKLALCISALCHDVDHPGLTNSFLVACNDPLAIRYNDLSVLENHHASITVKALTGYESMNILGIFPDSEQRHIRKLIVSCILATDMA    218
AcCAPE    58 LTLVEGQKMLNTLEVFALLLAALCHDVDHPGVNNAFLSITNDPLAIRYNDLSILESHHASTTIKTLLAYESTNILAPFSEAEQRHIRKLLVEIILATDMD    157
CbCAPE   198 LTQTELGDLLSSLEKYVAIISALCHDIDHPGYTNSFLILTNDRLAVRYNDRSVLESHHSATTFRVILDRKNATAIETFSKEEKALIRKLIVGTILATDMA    297
CoCAPE   119 LTQTEAGPLLTSLEKLAAMLGALCHDVDHPGLTNAFLVAAKDPLAIRYNDFAVLESHHAATMFKTMQERVEVDVLGQLSEGPRRHLRKLIVAIILATDMG    218

MpCAPE   275 EHARILREVGERVQDLRPFEQ-------------------------------------------SPFYTPPGCLSPILRDAESSSSGNTT----------    321
PpCAPE   199 VHAEIISSFTGRLNDRRPFEL-------------------------------------------SAPYTPPGPMPSKAPEPSDTGDTKTLRA--------    247
SmCAPE   219 YHGAVVQSFSLRSKEVGSFHA-------------------------------------------SSRYEYQPPPPPKKVPCKVTGIFEME----------    265
AcCAPE   158 HHFKIIEMLKSRSSDSNPFEC-------------------------------------------SPPYSMLDSR--HNSGTFPEGASSSS----------    202
CbCAPE   298 QHEDVLGALQARLVDTKPFQV-------------------------------------------SVPRRTEIMQEELSRQSNVAAAKGTASG--------    346
CoCAPE   219 QHSETVAAFAARMSDPRPFEQTVVAPPTPPQAAAAPAPAVTPPRTPQLKIPTSRAASLRPSPRPSPPHTARSTHSSDATSSQASTSKHVTDEGGSHRSDE    318

MpCAPE   322 --------------------------------------------------AGAKSSDAPP----SPKRLPDKVYKNPLSPSPP------IQSTSDVMLLI    361
PpCAPE   248 ------------------------------------------------DEASMSSSAAPPNDGNTNQPPPELKSPNPKYLLPP------LTSSSDVMLYM    293
SmCAPE   266 -----------------------------------------------------EQQEEPA--------------LLFPQVTKE------HRSSDDVLLLM    292
AcCAPE   203 -----------------------------------------------------QSSSGSQ--------------TTVVPSRPS------PSSTKDATLLL    229
CbCAPE   347 -------------------------------------------------LPKSDPSLAPS-------------VQTGTEFGERQETFYQSSATEDAILLL    384
CoCAPE   319 IQTPQDAPSEREERSSKGESIPSSRKGSQPPDKGVPRTPSRVSISRTQSVVTSAAVKGPAKTPRESRASNNFSQSPPATTVPPNPALHQTTSSTDALLLM    418

MpCAPE   362 QLIIKCADISNVVKPFFLSKRWAALLLLEWFRQGEIEKQLGLPISKFMDREDPSTLMAMTCGCIDYIAKPMYEVMTKLLPRMHENVLVNLNLNRQMWSTF    461
PpCAPE   294 QMIIKCADLSNVVKPFFLSKRWSALLLLEWFRQGDIERELGMRVSKFMERSEPTTLMDMTIGCIDFVAKPMFEATAVLLPHLHDEALANLTLNRSLWSSF    393
SmCAPE   293 KVIMKCSDISNTIRPYPLSKKWAALLLLEWFRQGDVERRLGMEISRNMNRNDPDSLQSMTLGCIDAFSIPMYEAVARVLPKLKNNVLPNLASNRESWMSF    392
AcCAPE   230 KMLIKCADISNVMKPFFLSKRWAALLLTEWFRQGDAEKKIGLPVSKNMDSENPVALQTMTLGCIDFLGEPMYETMALLLPKMHDEVLPNLEMNKSEWKSF    329
CbCAPE   385 KMLVKCADISNIIKPFFLSKRWAALLLIEWHRQGDTEKALGVPVSKFMDRDDPSTLIAMTCGCIDFIAEPMYIAMAGLAPKLKGVVQAHLSENRYLWEHY    484
CoCAPE   419 RMLIKCADISNLIKPFFLSKRWAALLLLEWFQQGDTEKRLGLPISKFMDREDSTTLMAMTCGCIDFIAKPMYETAARQIPALHTEAIPNMLTNRKQWMLF    518

MpCAPE   462 STNGRRASETAQQILGPFAPPPIPK--------------------------GEAVQEGYQQDHSKLEGKLVATFSGQYVSSDVPSRKLESSPSAKSLGSV    535
PpCAPE   394 SMGGRRGSEVARNILGPFLPPPIT-----------------------------------------IDG-IAEKFGEAAVKMDISSSSLHYD---------    442
SmCAPE   393 STAGP-IEQEVQVILGSFFP---------------------------------------------------IEGDDEQQRQSTSKSGARSS---------    431
AcCAPE   330 TSNGS-ALVTLEKFLSNFNS---------------------------------------------------EILQGKHPSLESSTSAPTME---------    368
CbCAPE   485 SMRYRRGSQEAALILGEHLPDLPPSKSFPQPRPEIERIKRRPRLASAATPMGGAYHLDPRVTEEQTPMLGVFQAGGSVRLIEWSSNNLPHTDDPSHQATP    584
CoCAPE   519 STNGRRGSATAADILGPLYP-----------------------------------------------------FGKRPDDDATSVGSIDMVP--------    557

MpCAPE   536 SEDSEETVENVEDSPILSVTDRSSEFSRLGSEAGSSVKTSPQFLSRAGTEAPSSPQSPLQASQEQRGSPSPGVQYRLSPRGPGSPSVPVTQISGNPESPG    635
PpCAPE   443 -------------------------------------------MYLPGVEKESLPMAPLQL---------------LPTLSPEEPTPLINELRMDEHHPS    484
SmCAPE   432 -----------------------------------------------QTGQSTMPES--------------------AQSSEGHRVSEIKKASVTT----    460
AcCAPE   369 -----------------------------------------------GQRHVTYGEG--------------------VGCSR-HVRNTMKSSGYSTSRDI    400
CbCAPE   585 DQVDDSDGGDLEVLRRAAG---GLPAIAEGSEHGKGVSRKEGVSSGSGTDAKNISPLQKAIAEIFPSSKGGMASLQRPSLSKDAGTSVIASSPLPQPLRP    681
CoCAPE   558 --------------------------------------------SELDLSLCATPAGG-----------------QFVPLAQTTMSAAPLSLLFGNKLRS    596

MpCAPE   636 YRETYGPGGSQPAAME----------VPFVQKSPHEPVAVDVDVAASQQSADVIAPLSKPQALQVKQPVVPTVSRGVHFDDSELTASERSELSSSIQSGS    725
PpCAPE   485 LLALDSSGDKSQSINK----------GPLRQ-----------DRESSILGG--AAPLS-----------LLRVNRNASLDAS---------------SGD    535
SmCAPE   461 -HTSPGNERPLSLLFG----------APLGR---------DVNTLASELAW---NTTLRD--------SSLRVNLVPSTRRS---------------HSS    514
AcCAPE   401 LETGKRTDRPLSLLVG----------GPLEE---------NSSTSRPRLSG---SKIVRDSVQSSSWDSSMRFDRSQVSKFR---------------EVS    463
CbCAPE   682 HGPSSAHGHAATSASQNFCPSVSSTVGSLWSRCASCCRAREANEIDPDQGGGSKETWDRSMSVNSSMSTNENTERGKRGFAAELVERIR-DISFRVRDIA    780
CoCAPE   597 ITDQGRRHTASAVARG----------MIMRS----------NSQLALELSEHSRGEKDRD----------HRGDNDNLFVTS----------------PR    650

MpCAPE   726 SPDLALRVSFVAINPDSDSVKVLIEQSDPLSTATIEQRDPDSPRMTGLEPFLEEVAASSSESKAEPETEDEYNQRYEAPLSLLFGAPTREELMLPAKIGK    825
PpCAPE   536 GNNLALR-SFVALRRN-----------------TVDERNRKPSSIS-----LPPPGYKGTEMMTDPS---------------------------------    579
SmCAPE   515 GSIGSLQ----LGPGQ---------------TVPS------------------ERGSSLAGTINDEM-------------------------------DY    546
AcCAPE   464 TTKLAFQPSKLIGHGK---------------KLPHPGRGMQSSFQLQSSPFPWERTALWEGSSTDKD-------------------------------GT    517
CbCAPE   781 ITHLAGEVMAEMNEDEISTIYSSNTSSFTGAEKSASTAAMVSLFSPKDSNRPEGISSKHKKELDPVTAALAPVEPSRTKAAGGPLTVAPESQVVASPIQP    880
CoCAPE   651 SRLGAFDTLAELNQGR----------------LLADG---------------VHKSALSADLNHETG---------------------------------    686

MpCAPE   826 DKNASLVSALLPGANNLVNDRKQNAWEQMAFRSPSEQAASRGSILTTVPSFANSDGDLEKSSSLRKGPPPGFWEALRTHPRVNRLNRALESKTWNALL-I    924
PpCAPE   580 -KNVALDIQSLAFIQDSAWDNRK------------------------------------------------FWDSIRRHPNGKQLHAFLESRLWISIN-I    629
SmCAPE   547 RHETSLSLDRIDVDKGPVG----------------------------------------------------FFTRLRARGVAIRVSQVLDSRVWAHFVAI    594
AcCAPE   518 KNHSPLS---IQIASHEIS----------------------------------------------------FWRILRMHPQAIKLNQFMDSKYWACLI-V    561
CbCAPE   881 KSWSALTSNQVRLSSQVSSQMTE-----------------------------------ENKADGKPKAKESFMDRVRQQPWAIKLEHIQNGTSWQVTM-I    944
CoCAPE   687 NRRPSHKMMSFRNIGRPRT----------------------------------------------------LLESIRESWWAVYVERFLNGTIMHTVM-L    733

MpCAPE   925 VATLVALFADDVVKGFLPKHADVYESHILTACLSLFLAESALLCIFRDRYFFSFFFWLDMLGSVSLVPLVIGITAQNLVIARTGRAAKTVTRFSKTLQAS   1024
PpCAPE   630 IATFVAMFANDFTKAILPKAG----------------------------YFLGFFFWLDLIGSVSLLTVITNVYTQNLVIARTGRAVKAMNRLSKSMQAT    701
SmCAPE   595 PATLIALFLNDFSRAFLPKSLDTFVDVVYVICLFLFLLDLVALSVLKPAYLFSFAFWQ------------------NLIVARVWKAAESLSRLAKKLQNR    676
AcCAPE   562 PAVVIALFSHNFIRAYCPKAADPYLIYIYLVCLALFIFELVALSIVRKDYFLGMSFWLDLVGSVTLIPLILGMLEINLIMARAAQAAKSVTRAAKWMQTT    661
CbCAPE   945 VCTLVGLFADDINQALFPKEYDKVVRGADFFCFFLFSFDLIAACIVRKGYFLSFFFFLDAVATISLLPVITDIFMKNLSIARAGRAARAGTRASRLLRFV   1044
CoCAPE   734 IATFCSLFSSDITIATLPKSWDPAETYILVWCLCTFILESILLSVFQVGYFFSFFFWLDMIATIALVPDITGLISTNLVIARAGRAARAGTRASRLLRFV    833

MpCAPE  1025 HIQQQIVHHIPVLRVFKFFG-FKRDSTLESPDYEEEEK-----FLSKPSQLWSRLAELTSQKLILGVLIIMILTPYFNNSEKDLAPLVSLDPLDDYLIGS   1118
PpCAPE   702 PLSS----MFPITYILKVFH-YKNVVVDDDEDEYFHVV-----SSTKPSQVWTELSELTAQKLIIGLLVMMIVSPLFRDNHRDLGPKMSLNTLDEFHGCT    791
SmCAPE   677 SAKVPSAFLIARLAKAVFYHCIRPLESKARDDKETTEDSSNQFMSSKPSYLWLRLSDLQSQRLVLGILILAIALPYFYQLPDDLAPTLALAALDSSPLYT    776
AcCAPE   662 DFSKSVIISTAKLFKRKY----KVQETEANKDIEPDPE---HHHKGKPSQVWTRLSELTSQKLVLGMLVLVILIPNLRIYTKDSGPISSLSMLDHSPLYS    754
CbCAPE  1045 RMAR-LLQLIKIVRAIKRRGLFRRRRSDDDGDDKDQSS---VIDSSKPSNVWRLLSELQTQKLIFGVLAMMIAVPLLHKDLVNLSPLASLSLLLEFPIFS   1140
CoCAPE   834 RLAR--VVNIGRITHSLWQP--HKGRMDAVHEFDEVDT-------SKPSNVWRMLSDLTTQKLIIGVMLMLIIMPLLRQERPDRGPPGTLTALDGLIFNT    922
```

Continued to the next page

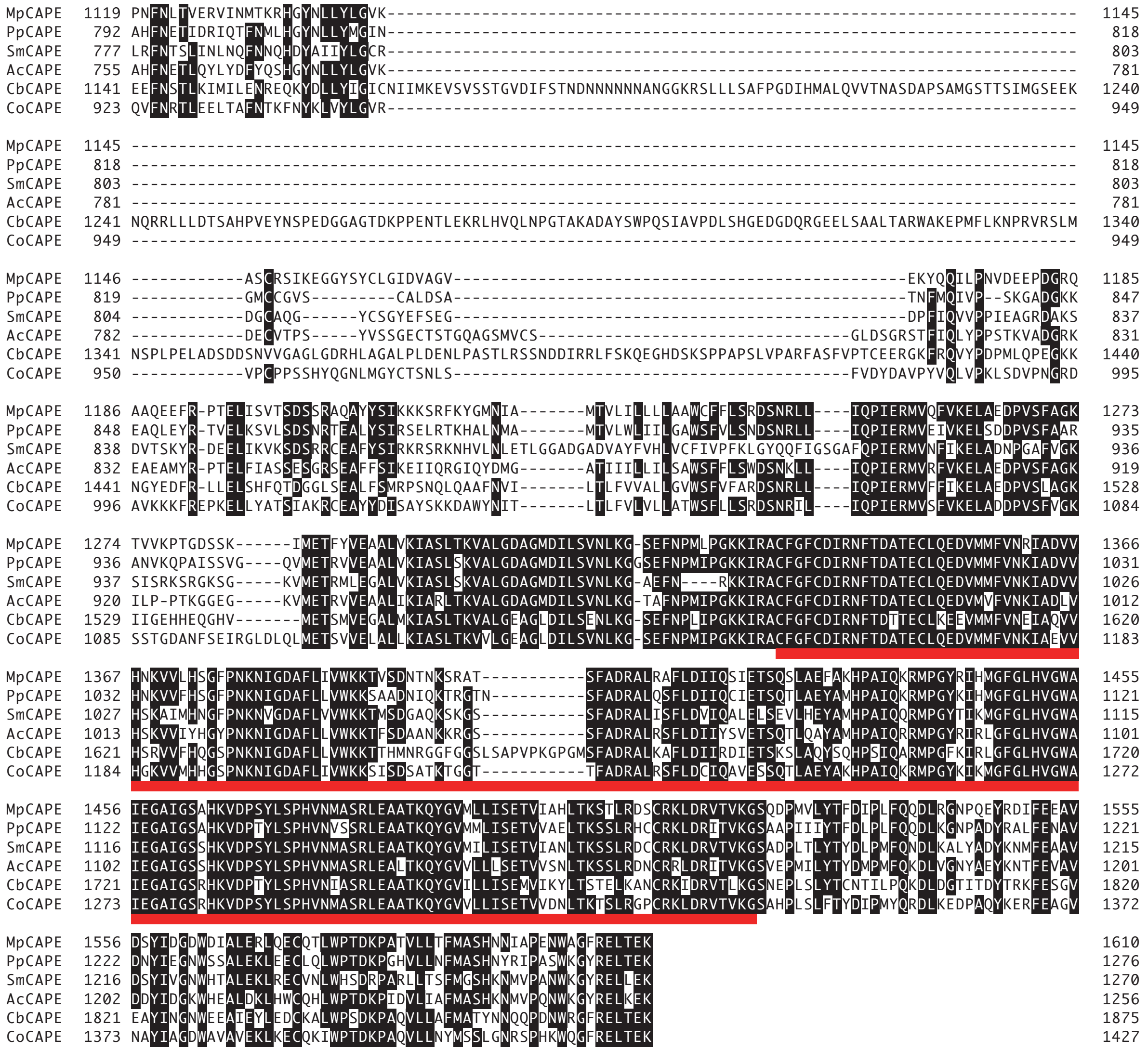

Figure S4. Alignment of MpCAPE with its orthologues. MpCAPE amino acid sequence is aligned with the orthologous sequences from *Physcomitrella patens* (PpCAPE), *Selaginella moellendorffii* (SmCAPE), *Adiantum capillus-veneris* (AcCAPE), *Coleochaete orbicularis* (CoCAPE) and *Chara braunii* (CbCAPE). Amino acid residues identical in majority of sequences are shown in black boxes. Gap introduced for good alignment are indicated by dashes. Number are amino acid positions for each protein sequence. Note that AcCAPE is a partial sequence. The catalytic domains of adenylyl cyclase (AC) and phosphodiesterase (PDE) are indicated by red and green bar, respectively.
